# Autonomic nervous regulation of ovarian function by noxious somatic afferent stimulation

**DOI:** 10.1007/s12576-014-0324-9

**Published:** 2014-06-26

**Authors:** Sae Uchida, Fusako Kagitani

**Affiliations:** Department of Autonomic Neuroscience, Tokyo Metropolitan Institute of Gerontology, 35-2 Sakaecho, Itabashi-ku, Tokyo, 173-0015 Japan

**Keywords:** Ovarian blood flow, Ovarian estradiol secretion, Ovarian sympathetic nerve, Noxious cutaneous stimulation, Reflex, Rat

## Abstract

It is well known that ovarian function is regulated by hypothalamic–pituitary–ovarian hormones. However, although several histological studies have described the autonomic innervation of the ovary, the involvement of these autonomic nerves in ovarian function is unclear. Recently, it has been shown that both the superior ovarian nerve (SON) and the ovarian nerve plexus (ONP) induce vasoconstrictor activity by activation of alpha 1-adrenoceptors, whereas the SON, but not the ONP, inhibits ovarian estradiol secretion by activation of alpha 2-adrenoceptors. Furthermore, reflex activation of these ovarian nerves by noxious cutaneous stimulation of the rat hindpaw results in ovarian vasoconstriction and inhibition of estradiol secretion. Thus, in addition to long-term regulation of ovarian function by hormones, ovarian autonomic innervation may be involved in rapid regulation of ovarian function by responding to either internal or external environmental changes.

## Introduction

Many studies have examined hypothalamic and pituitary hormonal regulation of such ovarian functions as ovulation and secretion of ovarian hormones [1, 2]. However, although several histological studies have described the innervation of the ovary by vagal parasympathetic and sympathetic nerves, including both afferents and efferents [3–8], the involvement of these autonomic nerves in ovarian function has not yet been clarified.

The activity of autonomic efferent nerves and function of the target organs are regulated reflexly by somatic afferent stimulation, for example noxious mechanical stimulation of the skin. The neural mechanism of reflex responses in the sympathetic and parasympathetic nervous systems produced by noxious somatic afferent stimulation has been described for anesthetized animals, for which emotional factors are eliminated [9, 10]. For example, cutaneous noxious mechanical stimulation of a hindpaw (pinching stimulation) increases heart rate and blood pressure via reflex activation of sympathetic efferent nerve activity to the heart and blood vessels [11]. The same somatic afferent stimulation increases adrenal sympathetic nerve activity and adrenal catecholamine secretion [12].

Concerning neural regulation of the female reproductive organs by autonomic nerves, some physiological functions have been demonstrated for afferents [13–16] and efferents [17] in the pelvic and hypogastric nerves innervating the uterus in rats. Furthermore, noxious somatic afferent stimulation has been demonstrated to cause both uterine contraction and an increase in uterine blood flow by reflex activation of the uterine parasympathetic efferent nerves of anesthetized rats [18].

Recently, a function of autonomic efferents innervating the ovary in rats has been demonstrated. These sympathetic efferents have vasoconstrictor activity in regulating ovarian blood flow and inhibitory activity in ovarian estradiol secretion [19–27]. Furthermore, these efferent sympathetic nerves to the ovary are activated by noxious cutaneous stimulation [19, 28].

Herein, we review the autonomic nervous regulation of ovarian function in anesthetized rats by noxious somatic afferent stimulation.

## Autonomic innervation of rat ovary

**Fig. 1:**
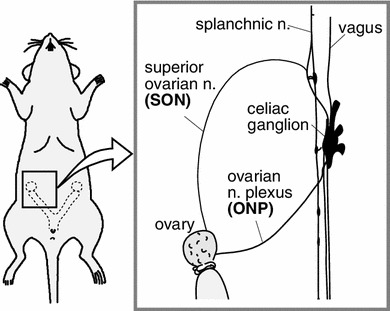
Schematic illustration of ovarian innervation by the ovarian nerve plexus (ONP) and the superior ovarian nerve (SON). (Modified from Kagitani et al. [23])

Histological studies have revealed the distribution and innervation of sympathetic (splanchnic) and parasympathetic (vagal) nerves of the rat ovary, including both afferents and efferents [3–8]. Sympathetic nerves innervating the ovary emerge from the lower thoracic and upper lumbar spinal cord segments (mainly at T9 and T10) whereas vagal nerves originate from medullary neurons in the nucleus of the solitary tract, the dorsal vagal complex, the nucleus ambiguus, and the area postrema [3, 7, 8]. These autonomic nerves reach the ovary by two routes: the ovarian nerve plexus (ONP) along the ovarian artery and the superior ovarian nerve (SON) in the suspensory ligament [29, 30] (Fig. 1). Histochemical and immunocytochemical studies have shown that the densities of nerves containing noradrenaline or neuropeptide-Y are high in the ovaries, whereas fewer nerves express acetylcholine, substance P, calcitonin gene-related peptide, vasoactive intestinal polypeptide, or other peptides [6]. Further, Burden et al. [3, 31] demonstrated that adrenergic nerves enter the ovary through the hilar perivascular plexus, and tiny branches from this plexus extend into the contiguous steroidogenic interstitial gland cells.

## Sympathetic regulation of ovarian blood flow

### Ovarian blood supply

The ovary receives blood from the ovarian and uterine arteries [32, 33]. The ovarian artery, which branches from the abdominal aorta or renal artery, crosses the ureter ventrally and runs to the ovary. The uterine artery runs along the uterine horn in the mesometrium and anastomoses with the ovarian artery before entry to the hilus of the ovary. In the ovarian hilus, the arteries divide into medullary arteries and then enter the ovarian cortex. These cortical arteries divide repeatedly in the cortex and supply the ovarian stroma, follicles, and corpora lutea [34].

**Fig. 2:**
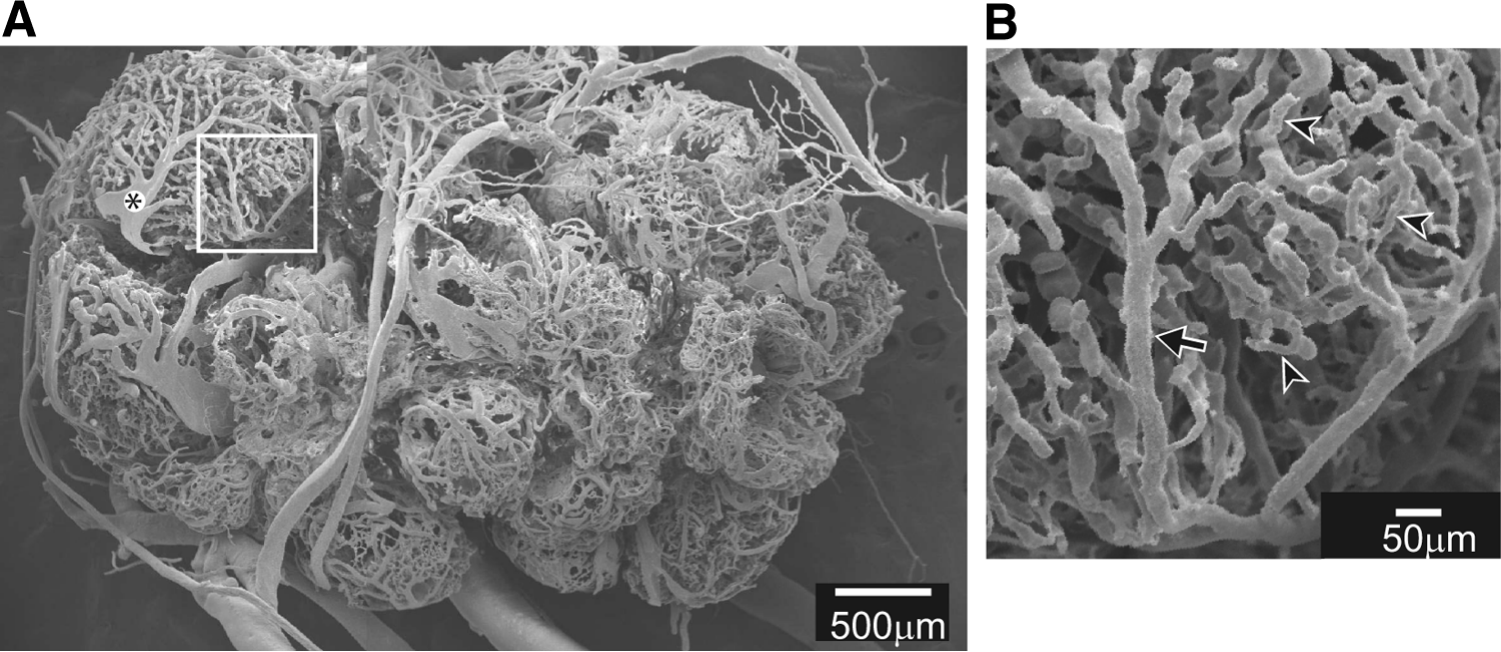
The vascular bed of a rat ovary. **a** Image of an ovarian vascular cast. **b** Enlarged image of the area framed in **a**. *Arrow*, arteriole; *arrowheads*, capillaries. (From Kanai et al. [21])

Figure 2a shows a vascular cast of a rat ovary, as observed with a scanning electron microscope [21]. Each ovarian follicle has a rich microvascular network. When examining the microvasculature on the surface of the ovary of anesthetized rats by video microscopy, arterioles and venules can be differentiated by their diameter, color of blood in the vessel, speed, and direction of blood flow. In the microvasculature shown in Fig. 2a, the arteriole (Fig. 2b arrow), venule (Fig. 2a*), and capillaries (for example, arrow heads in Fig. 2b) are distinguishable by reference to in-vivo observation. The diameter of ovarian arterioles on the surface of the vascular casts observed with a scanning electron microscope ranges from 11.4 to 34.3 μm (mean diameter, 21.9 ± 2.1 μm). This diameter is similar to that of ovarian arterioles on the surface of the ovary observed in vivo by video microscopy (range 13.1–42.9 μm; mean diameter, 22.1 ± 2.6 μm).

### Effect of electrical stimulation of ovarian nerves

Reynolds and Ford [35] described ovarian vasoconstrictor activity in in-vitro experiments with pig ovaries. A vasoconstrictive effect on ovarian blood vessels was attributed to the sympathetic neurotransmitter, noradrenaline (NA), in in-vitro experiments with human ovarian blood vessels [36] and in in-vivo experiments with rats [37, 38]. These earlier studies suggest the existence of adrenergic vasoconstrictor activity in the ovary.

**Fig. 3:**
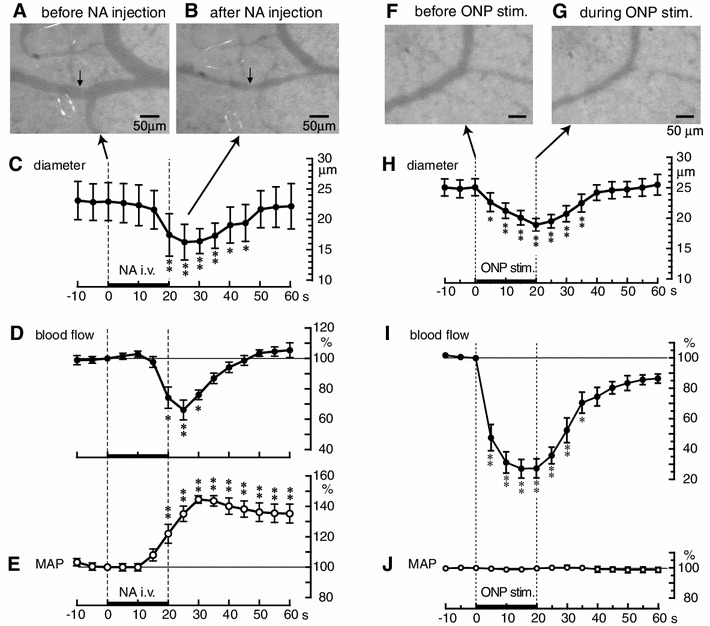
Effects of intravenous injection of noradrenaline (**a**–**e**) and of electrical stimulation of ONP (**f**–**j**) on the diameter of ovarian arterioles. **a**, **b**, **f**, **g**, Sample photographs of ovarian arterioles. **c**, **h**, Summary of changes in the diameters of ovarian arterioles. **d**, **e**, **i**, **j**, Summary of ovarian blood flow (**d**, **i**) and mean arterial pressure (MAP) (**e**, **j**) responses. Each *point and vertical bar* represents mean ± SEM. *p < 0.05, **p < 0.01; significantly different from the prestimulus control values, as defined by one-way repeated ANOVA followed by Dunnett’s multiple comparisons test. The *thin dashed vertical lines and the thick horizontal bars on the abscissae* indicate the time of noradrenaline (NA) injection at 5 μg/kg (**c**–**e**) or the time of electrical stimulation of the ONP at 10 V and 50 Hz for 20 s (**h**–**j**). (**a**–**e**, modified from Kanai et al. [21]; **f**–**j**, modified from Uchida et al. [22])

In anesthetized rats, we examined the effects of NA and electrical stimulation of the autonomic nerves to the ovary on the diameter of ovarian arterioles, by use of digital video microscopy, and on ovarian blood flow, by use of laser Doppler flowmetry [21, 22]. NA (5 μg/kg), injected intravenously into the jugular vein over 20 s resulted in a decrease in the diameter of ovarian arterioles and in ovarian blood flow, and an increase in mean arterial pressure (MAP) (Fig. 3a–e). The mean diameter of ovarian arterioles measured before NA injection was 23.0 ± 3.1 μm, but reached a minimum of 16.3 ± 2.9 μm 5 s after the end of the NA injection. Electrical stimulation of the distal part of the severed ONP resulted in a decrease in the diameter of ovarian arterioles and of ovarian blood flow, but did not change the MAP (Fig. 3f–j). The mean diameter of ovarian arterioles measured before ONP stimulation was 25 ± 1 μm, but reached a minimum of 19 ± 1 μm at the end of ONP stimulation. The time courses for the changes in ovarian blood flow were similar to those of the changes in the diameter of the ovarian arterioles. Electrical stimulation of the distal part of the severed SON also resulted in a decrease in ovarian blood flow (measured by plasma flow rate of ovarian venous blood) that was similar to that produced by ONP stimulation [23]. Decreases in ovarian blood flow after electrical stimulation of either the ONP or the SON were abolished completely by administration of an alpha-adrenoceptor antagonist [23, 24].

These results suggest that the activation of sympathetic nerves to the ovary (both the ONP and the SON) and NA, a sympathetic neurotransmitter, induce vasoconstriction of ovarian arterioles, thereby reducing blood supply to the ovaries.

## Sympathetic regulation of ovarian estradiol secretion

**Fig. 4:**
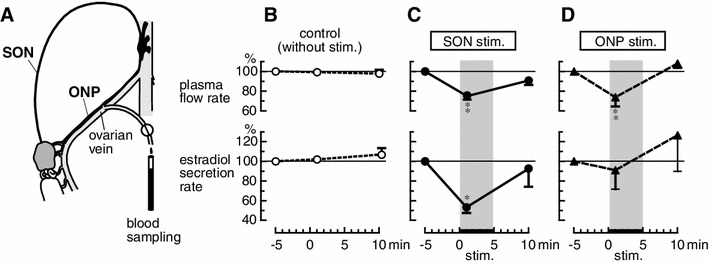
Ovarian venous plasma flow rate (*upper panels*) and rate of secretion of estradiol by the ovary (*lower panels*) after electrical stimulation of the SON and ONP. **a** Illustration showing the experimental procedures for collecting ovarian venous blood. **b** control condition (without stimulation). **c** SON stimulation. **d** ONP stimulation. Magnitudes of responses during and after stimulation are expressed as percentages of the prestimulus values (5 min before the stimulation). Each *point and vertical bar* represents the mean ± SEM. *p < 0.05; **p < 0.01; significantly different from the control response using two-way repeated ANOVA followed by Bonferroni post tests. (Modified from Kagitani et al. [23])

We examined the effects of electrical stimulation of the SON and the ONP on the rate of secretion of estradiol by the ovary in rats [23]. The rats were anesthetized on the day of estrus, and ovarian venous blood was collected intermittently through a catheter inserted into an ovarian vein (Fig. 4a). Plasma estradiol levels were measured by enzyme immunoassay. Under resting conditions, the mean concentration of estradiol in the ovarian venous plasma was 134.0 ± 13.3 pg/ml. The estradiol concentration in systemic arterial plasma (69.8 ± 6.9 pg/ml) was approximately 50 % of that observed in ovarian venous plasma. The rate of secretion of estradiol by the ovary was calculated from the absolute concentration of estradiol in the ovarian venous plasma minus the concentration in the arterial blood multiplied by the ovarian venous plasma flow rate. Under resting conditions, the ovarian venous plasma flow rate, concentration of estradiol in ovarian venous plasma, and the rate of secretion of estradiol ranged from 26.0 to 28.7 μl/min, 115.8 to 173.0 pg/ml, and 1.5 to 2.4 pg/min, respectively [23]. These values were stable for 45 min under resting conditions.

**Fig. 5:**
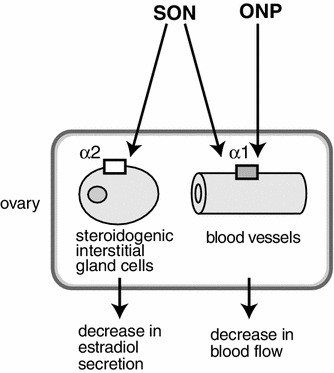
Schematic diagram of a possible mechanism for regulation of estradiol secretion by the ovary and blood flow of the ovary by stimulation of the SON and ONP. (Modified from Kagitani et al. [23])

The SON or ONP, ipsilateral to the ovary from which ovarian venous blood was collected, was stimulated electrically at an intensity which was supramaximum for C-fibers. Stimulation of either the SON or the ONP produced a decrease in ovarian venous plasma flow rate (Fig. 4c, d). During the SON or the ONP stimulation, reduction of the plasma flow rate reached 76 ± 3 % and 74 ± 9 % of the prestimulus values, respectively, and returned to the prestimulus basal level 5 min after the end of stimulation. On the other hand, the rate of secretion of estradiol by the ovary was reduced by SON stimulation but was not affected by ONP stimulation (Fig. 4c, d). During SON stimulation, reduction of the rate of secretion of estradiol reached 53 ± 6 % of the prestimulus values, and returned to the prestimulus basal level 5 min after the end of stimulation. These results suggest that activation of autonomic nerves to the ovary causes vasoconstriction and inhibition of estradiol secretion, independently (Fig. 5). Ovarian estradiol production is considered synonymous with release because steroid hormones, once produced, can freely cross the cell membrane without having to be packaged into granules and actively exocytosed [39]. Therefore, stimulation of the SON may reduce estradiol synthesis in the ovary.

Furthermore, it was shown that the reduction of estradiol secretion during SON stimulation was blocked by an alpha 2-adrenoceptor antagonist (yohimbine) but was not affected by an alpha 1-adrenoceptor antagonist (prazosin) or a beta-adrenoceptor antagonist (propranolol) [25]. On the other hand, reduction of ovarian blood flow during SON stimulation was blocked by an alpha 1-adrenoceptor antagonist but not affected by an alpha 2-adrenoceptor antagonist or a beta-adrenoceptor antagonist. These results indicate that decreases in ovarian estradiol secretion and ovarian blood flow in response to SON stimulation are caused by activation of alpha 2-adrenoceptors and alpha 1-adrenoceptors, respectively (Fig. 5).

Estradiol is synthesized from testosterone by aromatization in the ovary [40, 41]. Recently, we examined whether the inhibitory effect of SON on estradiol secretion via activation of alpha 2-adrenoceptors was a secondary response to an inhibitory effect of sympathetic nerve stimulation on testosterone synthesis. The rate of secretion of testosterone by the ovary was also reduced by electrical stimulation of the distal part of the severed SON. The reduction of the rate of testosterone secretion by SON stimulation was not affected by an alpha 2-adrenoceptor antagonist but it was abolished by an alpha 1-adrenoceptor antagonist. These results show that SON has an inhibitory role in ovarian testosterone secretion, via activation of alpha 1-adrenoceptors but not alpha 2-adrenoceptors [26, 27]. This, therefore, indicates that reduction of the rate of estradiol secretion by SON stimulation is because of direct inhibition of estradiol production.

Other examples of adrenergic innervation of endocrine secretory structures are found in the kidney [42] and pineal gland [43], where activation of beta-adrenoceptors increases secretion of renin and melatonin, respectively, and in the pancreas, where stimulation of alpha 2-adrenoceptors results in inhibition of insulin secretion [44–46].

## Regulation of ovarian blood flow and ovarian estradiol secretion by noxious somatic afferent stimulation

### Response of ovarian blood flow

**Fig. 6:**
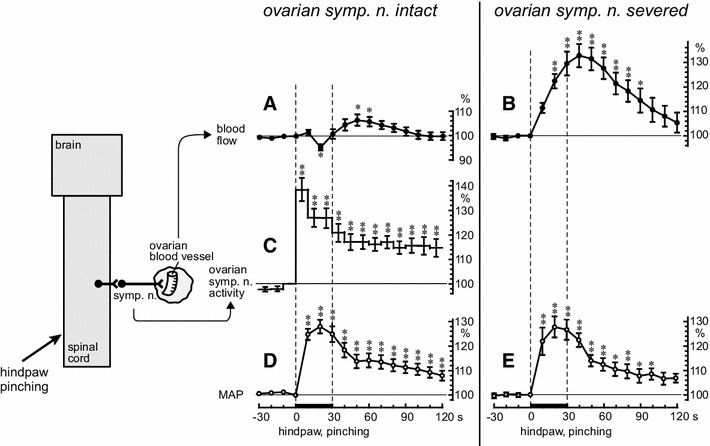
Summary of the responses of ovarian blood flow, activity of the ONP, and MAP to pinching of a hindpaw. **a**, **b**, **d**, **e**: Responses of ovarian blood flow (**a**, **b**) and MAP (**d**, **e**) under ovarian sympathetic nerves (ONP and SON)-intact (**a**, **d**) and severed (**b**, **e**) conditions. **c** Response of ONP activity. Ovarian blood flow, MAP, and nerve activity were calculated at 10-s intervals and were expressed as percentages of the prestimulus values (ordinates). The *thin dashed vertical lines* and the *thick horizontal bars* on the abscissa indicate the time of stimulation. Each *point and vertical bar* represents the mean ± SEM. The onset of pinching stimulation was set as time zero (abscissa). *p < 0.05; **p < 0.01, using one-way repeated ANOVA followed by Dunnett’s multiple comparison test. The schematic diagram on the left illustrates the experimental preparation. (Modified from Uchida et al. [20])

Effects of noxious mechanical stimulation of a hindpaw (pinching stimulation) on ovarian blood flow, ovarian sympathetic nerve (ONP) activity, and MAP have been examined in anesthetized rats [19, 20]. Pinching stimulation of a hindpaw for 30 s produced marked increases in ovarian sympathetic nerve activity and MAP (Fig. 6c, d). Ovarian blood flow decreased slightly (95 % of the prestimulus control level) during the stimulation and then slightly increased (106 % of the prestimulus control level) after stimulation (Fig. 6a).

After the ovarian sympathetic nerves (ONP and SON) were severed, pinching of a hindpaw was repeated. The MAP increased in the same way as before severing the ovarian sympathetic nerves (Fig. 6e) whereas a remarkable monophasic increase of the ovarian blood flow was observed (Fig. 6b). This increase in ovarian blood flow is explained by passive vasodilation because of a marked increase in MAP. When the ovarian sympathetic nerves are intact, the reflex increase in ovarian sympathetic nerve activity induced by pinching of a hindpaw may contribute to vasoconstriction of the ovarian blood vessels, and prevent an extreme passive increase in ovarian blood flow because of an increase in blood pressure [19, 20].

After spinal transection at the third thoracic segment, the responses to hindpaw stimulation of MAP, ovarian sympathetic nerve activity, and ovarian blood flow were nearly abolished. These results indicate that, in central nervous system-intact rats, hindpaw afferents contribute to a supraspinal reflex pathway to the ovarian sympathetic nerves innervating ovarian blood vessels (Fig. 8a).

### Response of ovarian estradiol secretion

**Fig. 7:**
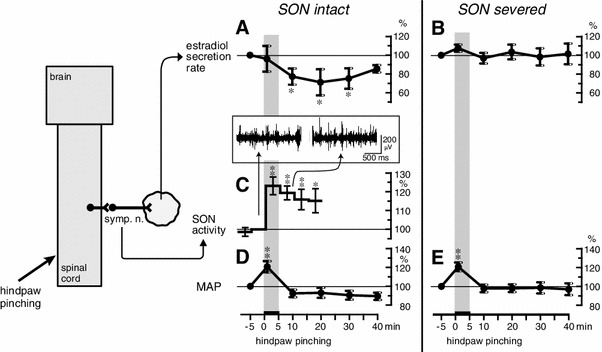
Summary of the responses: rate of ovarian estradiol secretion, activity of the SON, and MAP to pinching of a hindpaw. **a**, **b**, **d**, **e**: Rate of estradiol secretion (**a**, **b**) and MAP (**d**, **e**) for SON-intact (**a**, **d**) and severed (**b**, **e**) rats. **c** SON activity. Magnitudes of responses during and after stimulation are expressed as percentages of the prestimulus values (5 min before stimulation). Each *point and vertical bar* represents the mean ± SEM. *p < 0.05; **p < 0.01; significantly different from the prestimulus control values using one-way repeated-measures ANOVA followed by Dunnett’s multiple comparison test. *Inset* in **c**, sample recordings of SON activity before and after pinching stimulation. (Modified from Uchida et al. [28])

**Fig. 8:**
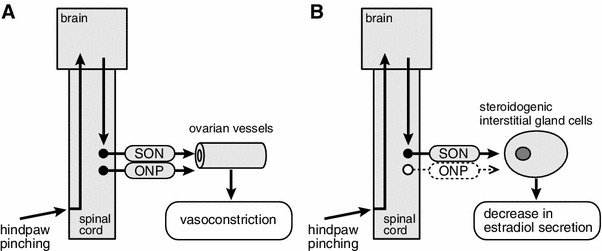
Schematic diagrams of the reflex pathway for ovarian blood flow (**a**) and estradiol secretion (**b**) responses elicited by hindpaw pinching

It has been shown for anesthetized rats that noxious mechanical stimulation (pinching) of a hindpaw for 5 min produced an increase in SON activity and a decrease in the rate of secretion of estradiol by the ovary (Fig. 7a, c) [28]. The increase in SON activity reached maximum level during stimulation, and the activity level remained elevated for more than 15 min after the termination of stimulation. Reduction of the rate of estradiol secretion reached significance by 5 min after the end of stimulation and lasted for 20 min. The rate of estradiol secretion decreased to 71 % of prestimulus basal values 15 min after the stimulation ended. The decrease in the rate of ovarian estradiol secretion was abolished by bilateral transection of the SON (Fig. 7b). Mean arterial pressure was increased only during stimulation, and the MAP response was not affected by severing the SON (Fig. 7d, e). These results suggest that the decrease in estradiol secretion in response to noxious mechanical stimulation of a hindpaw may be a consequence of the reflex increase in SON activity (Fig. 8b). After spinal transection at the second cervical level, the increased SON activity in response to hindpaw pinching was abolished. This indicates that the reflex center for the increase in SON activity in response to hindpaw pinching is located in supraspinal structures.

Pinching stimulation of a hindpaw for 5 min reduced the rate of estradiol secretion by the ovary, but did not change the plasma estradiol concentration in systemic blood [28]. The clinical implication of this work is that ovarian dysfunction because of activation of sympathetic nerves to the ovary does not immediately result in changes in systemic blood. This may be one reason why some ovarian diseases are difficult to detect clinically at an early stage.

## Conclusion

We have reviewed several interesting results from recent studies. First, ovarian blood flow and ovarian estradiol secretion are controlled independently by sympathetic adrenergic innervation. Of the two pathways of sympathetic nerves to the ovary (SON and ONP), stimulation of either reduces ovarian blood flow via activation of alpha 1-adrenoceptors, whereas stimulation of the SON, but not the ONP, reduces estradiol secretion via activation of alpha 2-adrenoceptors. Second, reflex activation of ovarian sympathetic nerves by noxious cutaneous stimulation causes ovarian vasoconstriction and inhibition of ovarian estradiol secretion. The ovarian vasoconstrictive response is produced via reflex activation of the SON and ONP, whereas inhibition of ovarian estradiol secretion occurs as a result of reflex activation of the SON only (Fig. 8). These results suggest that reflex activation of sympathetic nerves to the ovary by stressful physical stimulation, for example noxious stimulation, may be involved in rapid inhibition of ovarian function in emergencies. Rapid and direct regulation of ovarian function by the autonomic nerves may be an important adaptation of female reproductive function to either internal or external environmental changes. This is in addition to the long-term regulation provided by hypothalamic–pituitary hormones. It is also possible that hyperactivity of sympathetic nerves to the ovary may contribute to ovarian failure, for example anovulation, by eliciting ovarian vasoconstriction and reduction of ovarian estradiol secretion [33, 47, 48]. These findings extend our understanding of neural regulation of ovarian function, which occurs in addition to hormonal regulation by the hypothalamic–pituitary–ovarian axis.
